# Involvement of polymorphisms of the nerve growth factor and its receptor encoding genes in the etiopathogenesis of ischemic stroke

**DOI:** 10.1186/s12881-018-0551-7

**Published:** 2018-03-02

**Authors:** Ani Stepanyan, Roksana Zakharyan, Arsen Simonyan, Gohar Tsakanova, Arsen Arakelyan

**Affiliations:** 1 Institute of Molecular Biology NAS RA, 7 Hasratyan Str, 0014 Yerevan, Armenia; 2 Hospital and Polyclinic №2 CJSC, 54 Aram Str, 0002 Yerevan, Armenia

**Keywords:** Ischemic stroke, Nerve growth factor, Nerve growth factor receptor, NGF, NGFR, Single nucleotide polymorphism

## Abstract

**Background:**

Despite the important role of the nerve growth factor in the survival and maintenance of neurons in ischemic stroke, data regarding the relationships between variations in the encoding gene and stroke are lacking. In the present study, we evaluated the association of the functional polymorphisms in *NGF* (rs6330) and *NGFR* (rs2072446 and rs734194) genes with ischemic stroke in an Armenian population.

**Methods:**

In total, 370 unrelated individuals of Armenian nationality were enrolled in this study. Genomic DNA samples of patients and healthy controls were genotyped using polymerase chain reaction with sequence-specific primers.

**Results:**

The results obtained indicate that the minor allele of rs6330 (*P*_*corr*_ = 2.4E-10) and rs2072446 (*P*_*corr*_ = 0.02) are significantly overrepresented in stroke group, while the minor allele of rs734194 (*P*_*corr*_ = 8.5E-10) was underrepresented in diseased subjects. Single nucleotide polymorphisms in *NGF* gene (rs6330) and *NGFR* gene (rs2072446 and rs734194) are associated with the disease. Furthermore, it was shown that the carriage of the *NGF* rs6330*T minor allele is associated with increased infarct volume and higher risk of recurrent stroke.

**Conclusions:**

In conclusion, our findings suggest that the *NGF* rs6330*T and *NGFR* rs2072446*T minor alleles might be nominated as a risk factor for developing ischemic stroke and *NGFR* rs734194*G minor allele as a protective against this disease at least in Armenian population.

## Background

According to the World Health Organization, 15 million people suffer stroke worldwide each year, six million of them die and five million remain partially or completely disabled. Stroke is the second leading cause of disability and death above the age of 60 years, and the fifth leading cause of death in people aged 15 to 59 years old [1]. Stroke problem is urgent for developing world, because the incidence of this disease is increasing. Thus, according to the last data published by Ministry of Health of Armenia, in 2015 there were approximately new 768 cases of cerebrovascular disease incidence and 81 lethal cases per 100,000 general population of Armenia [2].

**Fig. 1:**
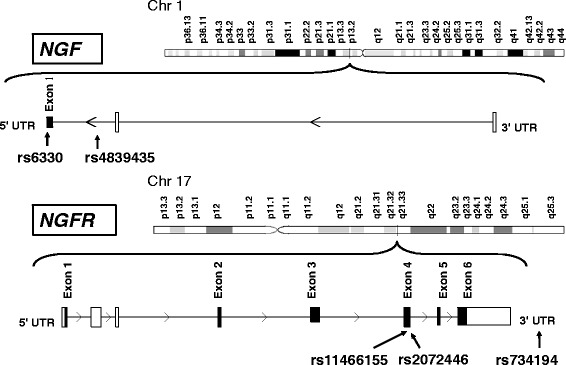
The localization of the selected 5 SNPs in *NGF* gene and *NGFR* gene

Growing evidence suggests that nerve growth factor (NGF) besides its neurotrophic function has sufficient influence on inflammatory and vascular endothelial cells [3, 4]. Previous studies demonstrated that altered presence of NGF and its receptor (NGFR) are linked to several important processes such as vascular hypertension [5], atherogenesis [6] and inflammation [7] which are considered to be risk factors for the development of ischemic stroke (IS). Moreover, NGF plays an important role in the processes of neuronal survival, ischemic tolerance of the brain and it is involved in the mechanism by which neurons can be protected from cell death. Likewise, it was found that intraventricular NGF ameliorated the development of delayed neuronal death [8]. NGF is a known activator for ERK5 (mitogen-activated protein kinase 5) which is key modulator of neuroprotection [9]. Furthermore, NGF up-regulates the expression of the KLF4 transcription factor that plays important role in initiating an anti-apoptotic and anti-inflammatory response [10]. Thus, recent studies suggest ERK5/KLF4 cascade as a common downstream signaling pathway for NGF-induced neuroprotection against oxidative stress [11]. Hence NGF is involved in several crucial processes related to IS etiopathogenesis and it is a good candidate to influence the susceptibility to IS, which is considered to be a complex polygenic disease [12]. There are number of single nucleotide polymorphisms (SNPs) in genes encoding NGF and NGFR that may alter their function and expression levels [13]. Thus, we have used a candidate polymorphism study approach to identify the possible association of the several functional SNPs (Fig. 1) in the genes encoding NGF (rs6330 and rs4839435) and NGFR (rs11466155, rs2072446 and rs734194) with IS in Armenian population. These polymorphisms were selected based on previous studies implicating them in several neurodegenerative, psychological, neurological and neurovascular diseases [13–15].

## Methods

### Subjects

In total, 170 patients with IS (males/females: 88/82; mean age ± SD: 55 ± 9.7 years) and 200 healthy subjects (males/females: 109/91; mean age ± SD: 49 ± 9.2 years) were enrolled in this study. All subjects were unrelated Caucasians of Armenian ancestry.

Patients were hospitalized in the neurological departments of medical centers of the Ministry of Health of Republic of Armenia (MH RA). Diagnosis of IS was based on clinical history and neurological examination of patients and was confirmed by brain computer tomography (CT) imaging and basal laboratory tests. According to the CT used for the quantitative assessment of the volume of the ischemic area, in 42.5% of IS patients the volume of infarct area was less than 60cm^3^ and in 57.5% of patients the volume of infarct area was more than 60cm^3^. Stroke subtype was classified according to TOAST definitions [16]. Stroke severity was scored using the National Institutes of Health Stroke Scale [17]. Among IS patients involved in this study 38 had cardioembolic stroke and 132 had atherothrombic stroke. Sixty six patients presented anatomically relevant CT hypodense areas in cortical-subcortical parts of cerebral right hemisphere, 97 in left hemisphere and 7 in brain stem. Among IS patients 112 had hyperlipidemia, 87 had arterial hypertension, 40 had atrial fibrillation and 66 had coronary artery disease. With regard to the lifestyle habits 110 patients were current smokers, and 59 were alcohol consumers. Seventy eight patients had positive family history of IS, including 48 maternal, 27 paternal and 3 biparental inheritance.

Healthy subjects (controls) without family history of IS and myocardial infarction were recruited among the blood donors of the Erebouni Medical Center of the MH RA and Blood Bank of Haematology Center after prof. R.Yeolyan. Healthy controls had no serious medical disorders, including coronary artery disease, atrial fibrillation, arterial hypertension, and hyperlipidemia, or treatment during the past 12 months. No special studies have been performed to assess the progress of atherosclerotic process in healthy subjects group. Exclusion criteria for all subjects included past or present history of neuropsychiatric metabolic disorders, myocardial infarction, as well as oncological and immune system diseases.

### Collection of blood samples and extraction of genomic DNA

About 5 ml of venous blood was collected from each study subject by venipuncture in EDTA-containing tubes. Blood samples of IS patients were collected on days 1–4 of stroke onset. Genomic DNA was isolated from fresh blood samples according to the standard phenol-chloroform method and stored at − 30 °C until further use [18].

### Selection of the SNPs in *NGF* and *NGFR* genes

According to the National Center of Biotechnology Information (NCBI) databases [http://www.ncbi.nlm.nih.gov/], from 6132 SNPs found in *NGF* gene and from 2714 SNPs in *NGFR* gene the 120 and 178 SNPs are non-synonymous, respectively. From these SNPs we selected those which: 1) have MAFs > 0.05; 2) have reported association with several neurological and neurovascular diseases; 3) presumably have influence on structure and/or function of NGF and NGFR proteins and 4) were linked to altered protein levels in serum [13, 19–26].

### Genotyping of selected SNPs in *NGF* and *NGFR* genes

All DNA samples were genotyped for selected SNPs using polymerase chain reaction with sequence specific primers (PCR-SSP) using earlier described conditions [13, 18]. Detailed characterizations of the *NGF* rs6330 and rs4839435*,* as well as *NGFR* rs11466155, rs2072446 and rs734194 SNPs are shown in Fig. 1 and Table 2. All primers for the PCR-SSP were designed using the reference genomic sequences in the GenBank (http://www.ncbi.nlm.nih.gov). The primer sequences designed for mentioned SNPs and amplicons length are shown in Table 1.

**Table 1:** The primer sequences and amplicons size of the studied SNPs

Gene	Accession	SNP ID	Primer type	Nucleotide sequence of primer (5′ → 3′)	Amplicon size, bp
*NGF*	NG_007944	rs6330	reverse for standard C allele	CTGAAGTTTAGTCCAGTGGG	187
			reverse for minor T allele	CTGAAGTTTAGTCCAGTGGA	
			forward constant	CTGCATTTAGTACTCCATGAA	
		rs4839435	forward for standard G allele	TGGGTGCCAAAAAGCTTGGC	188
			forward for minor A allele	TGGGTGCCAAAAAGCTTGGT	
			reverse constant	GCAGCTCCTGCAATTATCCA	
*NGFR*	AC006487	rs11466155	reverse for standard C allele	AGGCTATGTAGGCCACAAGG	210
			reverse for minor T allele	AGGCTATGTAGGCCACAAGA	
			forward constant	CAGAGGGCTCGGACAGCACA	
		rs2072446	forward for standard C allele	GTCCACACCCCCAGAGGGCTC	190
			forward for minor T allele	GTCCACACCCCCAGAGGGCTT	
			reverse constant	AGCAGCCAGGATGGAGCAAT	
		rs734194	forward for standard T allele	GCTGGAGCTGGCGTCTGTCT	186
			forward for minor G allele	GCTGGAGCTGGCGTCTGTCG	
			reverse constant	CTAGAGCTGGGAGAAATCCC	

The presence/absence of allele-specific amplicons in PCR products was visualized by electrophoresis in 2% agarose gel stained with ethidium bromide.

### Statistical analysis

Distribution of genotypes for the mentioned SNPs was checked for correspondence to Hardy-Weinberg equilibrium (HWE) by Pearson χ^2^ test. To reveal a potential association of these SNPs with IS, genotype, allele, and phenotype frequencies (carriage rates) in patients and controls were compared. The significance of differences between study groups in multiplicative, dominant and recessive models was determined using a Fisher’s exact test. With regard to additive model the differences in genotype distribution of the polymorphisms between case and control subjects were tested by logistic regression. The odds ratio (OR), 95% confidence interval (CI), and exact *P* value (*P*_*nom*_) were calculated. Nominal *P* values (*P*_*nom*_) were adjusted for multiple testing by Bonferroni correction (factor 5). Corrected *P* values (*P*_*corr*_) < 0.05 were considered significant [27]. The Mann-Whitney U (quantitative variables) and Fisher’s exact test (nominal and ordinal variables) were used to evaluate the possible differences of the clinical characteristics between minor allele carrier and non-carriers IS patients.

## Results

The genotyping analysis showed that genotype distribution of the *NGF* gene rs6330 and rs4839435 and the *NGFR* gene rs11466155, rs2072446 and rs734194 SNPs in study groups were concordant with HWE (*P* > 0.05) (Table 2). Furthermore, the observed minor allele frequencies (MAFs) of the studied SNPs (Table 2) in Armenian population were compared from those reported in major (*n* = 2504) population of the 1000 Genomes Project. The results showed significant difference between MAFs of the *NGF* gene rs4839435 as well as *NGFR* gene rs2072446 and rs734194 SNPs in Armenian population compared to 1000 Genomes Project data, whereas there was no difference for other two studied SNPs (*NGF* gene rs6330 and *NGFR* gene rs11466155) (Table 2). SNP analysis revealed that rs6330 variation of the *NGF* gene is associated with IS in the multiplicative model (*P*_*corr*_ = 3.6E-11), the dominant model (*P*_*corr*_ = 2.4E-10), the recessive model (*P*_*corr*_ = 0.001), and the additive model (*P*_*corr*_ = 0.0005) (Table 3). Moreover, the frequency and carriage (dominant model) of *NGF* rs6330*T minor allele were 1.98 (0.406 vs. 0.205; *P*_*corr*_ = 3.6E-11) and 1.85 (0.647 vs. 0.35; *P*_*corr*_ = 2.4E-10) times respectively increased in IS patients in comparison with healthy controls (Table 3). In IS patients group, Mann-Whitney U test revealed statistically significant difference of the volumes of ischemic area between *NGF* gene rs6330*T minor allele carriers and non-carriers (CT + TT vs. CC; 188 ± 234 cm^3^ vs. 72 ± 128 cm^3^; *P* = 0.023). Furthermore, 27% of all patients enrolled in study experienced second or third IS; and also there were significantly higher number of recurrent IS events in T minor allele carriers compared to non-carriers (CT + TT vs. CC, 39.3% vs. 10.5%; OR = 5.71; 95% CI: 2.68–12.2; *P* = 1.0E-6).

**Table 2:** HWE and MAFs of the selected SNPs in the studied groups and in major population

Gene SNP ID	Minor allele	MAF (frequency/number)			HWE (*P*)	
		IS patients	Controls	1000 Genomes	IS patients	Controls
*NGF* rs6330	T	0.406/138	0.205/82	0.248/1239	0.95	0.12
			*P* = 0.06			
*NGF* rs4839435	A	0.344/117	0.333/133	0.176/880	0.98	0.165
			*P* < 0.0001			
*NGFR* rs11466155	T	0.282/96	0.263/102	0.229/1151	0.5	0.6
			*P* = 0.25			
*NGFR* rs2072446	T	0.378/125	0.293/117	0.053/264	0.99	0.094
			*P* < 0.0001			
*NGFR* rs734194	G	0.1/34	0.273/109	0.106/533	0.72	0.26
			*P* < 0.0001			

*MAF* minor allele frequency, *HWE* Hardy–Weinberg equilibrium

**Table 3:** Association of the *NGF* and *NGFR* genes SNPs with IS risk under different models

Gene SNP (M/m)	Groups	Genotypes (number/frequency)			Model	OR (95%CI)	*P*_*nom*_
		MM	Mm	mm			
*NGF* rs6330 (C/T)	IS patients	60 (0.35)	82 (0.48)	28 (0.175)	multiplicative	2.65 (1.996–3.5)	7.3E-12
					dominant	3.39 (2.35–4.89)	4.8E-11
	Controls	130 (0.65)	58 (0.29)	12 (0.06)	recessive	3.07 (1.65–5.72)	0.0002
					additive	3.409 (2.3–5.054)	< 0.0001
*NGF* rs4839435 (G/A)	IS patients	73 (0.43)	77 (0.45)	20 (0.12)	multiplicative	1.05 (0.81–1.37)	0.45
					dominant	0.98 (0.69–1.4)	1
	Controls	85 (0.425)	97 (0.485)	18 (0.09)	recessive	1.35 (0.76–2.39)	0.3
					additive	0.851 (0.58–1.24)	0.4
*NGFR* rs11466155 (C/T)	IS patients	86 (0.505)	72 (0.424)	12 (0.071)	multiplicative	1.11 (0.84–1.46)	0.48
					dominant	1.19 (0.84–1.69)	0.32
	Controls	110 (0.55)	75 (0.375)	15 (0.075)	recessive	1.06 (0.54–2.07)	0.86
					additive	1.791 (1.15–2.79)	0.011
*NGFR* rs2072446 (C/T)	IS patients	68 (0.4)	79 (0.465)	23 (0.135)	multiplicative	1.43 (1.09–1.86)	0.008
					dominant	1.68 (1.18–2.39)	0.004
	Controls	105 (0.525)	73 (0.365)	22 (0.11)	recessive	1.3 (0.76–2.23)	0.3
					additive	2.327 (1.56–3.48)	< 0.0001
*NGFR* rs734194 (T/G)	IS patients	138 (0.81)	30 (0.18)	2 (0.01)	multiplicative	3.36 (2.36–4.78)	2.3E-12
					dominant	3.62 (2.42–5.42)	1.7E-10
	Controls	109 (0.545)	73 (0.365)	18 (0.09)	recessive	7.95 (2.35–26.89)	9.7E-5
					additive	0.182 (0.11–0.32)	< 0.0001

“M/m” indicates major/minor alleles; multiplicative model indicates “M vs. m”; dominant model indicates “Mm + mm vs. MM”; recessive model indicates “MM + Mm vs. mm”; additive model indicates “MM vs. Mm vs. mm”

According to the data obtained for the genotyping of the *NGFR* gene rs2072446 SNP*,* the frequency and carriage (multiplicative and dominant models) of T minor allele were 1.29 (0.378 vs. 0.293; *P*_*corr*_ = 0.04) and 1.26 (0.6 vs. 0.475; *P*_*corr*_ = 0.02) times respectively increased in IS patients compared to controls. Further, there were statistically significant difference of the frequencies of the rs2072446 polymorphism CC, CT and TT genotypes (additive model) between patients and controls groups (*P*_*corr*_ = 0.0005).

A strong negative association was detected between IS and *NGFR* gene rs734194 genetic variation in the all studied models (multiplicative *P*_*corr*_ = 1.1E-11; dominant *P*_*corr*_ = 8.5E-10; recessive *P*_*corr*_ = 4.8E-4; additive *P*_*corr*_ = 0.0005) (Table 3). Particularly, *NGFR* rs734194*G minor allele frequency and carriage were 2.73 (0.1 vs. 0.273; *P*_*corr*_ = 1.1E-11) and 2.42 (0.188 vs. 0.455; *P*_*corr*_ = 8.5E-10) times respectively decreased in IS patients in comparison with controls.

Finally, no association with IS were observed for rs4839435 SNP of the *NGF* gene and the rs11466155 SNP of the *NGFR* genes (Table 3).

According to LD analysis results (Table 4) *NGF* gene rs6330 and rs4839435, as well as *NGFR* gene rs11466155, rs2072446 and rs734194 SNPs are not in strong LD among all studied groups. Furthermore, LD between selected SNPs in *NGFR* gene in Armenian population differs from LD in population samples from the HapMap Project [28]. As for obtained linkage between *NGF* gene rs6330 and rs4839435 SNPs in Armenian population it is almost the same as in HapMap (Table 4).

**Table 4:** LD values (the absolute D’’ and r^2^) IS patients (b), and r^2^ values calculated from the data obtained for Armenian population involved in this study and for the results available on HapMap (c)

(a)					
r^2^/|D'|	*NGF* rs6330	*NGF* rs4839435	*NGFR* rs11466155	*NGFR* rs2072446	*NGFR* rs734194
*NGF* rs6330	–	0.01867	–	–	–
*NGF* rs4839435	0.18280	–	–	–	–
*NGFR* rs11466155	–	–	–	0.01859	0.10206
*NGFR* rs2072446	–	–	0.14499	–	0.01801
*NGFR* rs734194	–	–	0.34901	0.13786	–
(b)					
r^2^/|D'|	*NGF* rs6330	*NGF* rs4839435	*NGFR* rs11466155	*NGFR* rs2072446	*NGFR* rs734194
*NGF* rs6330	–	0.06598	–	–	–
*NGF* rs4839435	0.28271	–	–	–	–
*NGFR* rs11466155	–	–	–	0.12024	0.07141
*NGFR* rs2072446	–	–	0.41654	–	0.08063
*NGFR* rs734194	–	–	0.49926	0.63728	–
(c)					
Armenians/HapMap	*NGF* rs6330	*NGF* rs4839435	*NGFR* rs11466155	*NGFR* rs2072446	*NGFR* rs734194
*NGF* rs6330	–	0.01867	–	–	–
*NGF* rs4839435	0.012	–	–	–	–
*NGFR* rs11466155	–	–	–	0.01859	0.10206
*NGFR* rs2072446	–	–	0.001	–	0.01801
*NGFR* rs734194	–	–	0.002	0.609	–

For each pair of SNPs r^2^ and |D’’| values are shown above and below the dioganal, respectively

In order to evaluate an interaction between the significant alleles at the two loci (*NGF* rs6330/*NGFR* rs2072446) we compared the relative risk of IS in patients carrying one of the risk alleles with those who have two risk alleles at both loci. Increased relative risk for IS was observed in double risk allele carrier patients compared to carriers of either *NGF* rs6330*T or *NGFR* rs2072446*T alleles (Table 5).

**Table 5:** Relative risk of IS among carriers of *NGF* rs6330*T, *NGFR* rs2072446*T and TT (rs6330/rs2072446) alleles

	*NGF* rs6330*T	*NGFR* rs2072446*T	TT(rs6330/rs2072446)
Relative risk	1.85	1.26	2.16
95% CI	1.48–2.3	1.05–1.53	1.46–3.17
Significance level	*P* < 0.0001	*P* = 0.001	*P* = 0.0001

## Discussion

In the current study, we observed positive association of the *NGF* gene rs6330 and *NGFR* gene rs2072446 SNPs with IS. In contrast, rs734194 genetic variant of the *NGFR* gene showed negative association with IS susceptibility. We have also observed that the carriage of the T minor allele of *NGF* gene rs6330 polymorphism is associated with increased infarct volume and higher risk of recurrent IS.

Recent GWAS reported number of loci which are associated with IS risk [29–31]. Despite the populations included in these studies were almost the same, still differences in reported results were present. Thus the study by Ikram et al. [31] identified two intergenic SNPs within 11 kb of the gene *NINJ2* associated with IS. Two recent GWAS studies [29, 30] did not replicate this finding, however, partially confirmed several loci reported from other studies [32, 33] as well as identified new ones. Despite the fact that latter studies were performed by same consortium, only four loci were overlapping. The general disparity of the results might be partly explained by different quality control filters before imputation as well as methods for assessing population structure in GWAS or due to genetic differences between populations included in these studies. For example, an association of the *PRKCH* locus with stroke identified in a GWAS in Japanese participants was not found in European populations [30].

Genomic loci that are associated with increased stroke susceptibility and were reported by recent GWAS in European, non-Hispanic black and Hispanic ancestry samples do not include *NGF* and *NGFR* genes [29, 30]. Inconsistency of our data with the published results might be explained due to a long period of isolation of Armenian population since the Bronze Age and subsequently a unique profile of rare disease alleles [34]. Furthermore, recent studies reported very restricted genetic affinities of Armenians with European populations [35, 36]. From the other side the genetic structure of Armenian population is still largely unknown, which forces as to employ candidate gene approach to study *NGF* and *NGFR*, since their involvement in neuroprotection after IS and several pathogenic processes contributing to the development of IS is well documented [5–9].

*NGF* rs6330 and *NGFR* rs2072446 functional SNPs lead to non-synonymous amino acid substitutions and are possibly involved in the gene expression and protein secretion [13, 19]. The first one is described as a C104 → T exchange in exon 1 of the NGF gene and leads to amino acid substitution of alanine to valine at position 35 (Ala35Val). Some authors suppose that the increase in amino acid size at this position could modify the tertiary structure of NGF, leading to altered interaction and signaling via the NGFR [20]. Therefore, T minor allele could be a risk factor for IS through its possible involvement in increased cell death and consequently, decreased neuronal survival after ischemia. This is consistent with our findings on association of rs6330*T minor allele with increased lesion volume after IS. With regard to *NGFR* rs2072446 polymorphism, the C739 → T nucleotide transition in exon 4 leads to serine to leucine substitution at the amino acid position 205 (Ser205Leu) and the change of polar (hydrophilic) amino acid to nonpolar (hydrophobic) subsequently, causes alteration of the NGFR protein structure and function [21]. In addition, rs734194 is located on 3’ UTR and thereby it could be engaged in regulating the mRNA stability and translational efficiency. Hence, it is possible that this SNP alters the expression of NGFR or the binding of NGF, which can decrease neuronal apoptosis rate [22]. All together, these mechanisms may partially explain the involvement of the *NGF* rs6330 and the *NGFR* rs2072446 in the higher risk of IS, and the protective role of *NGFR* rs734194 against this disease observed in our study.

Recent studies reported that rs6330, rs2072446 and rs734194 genetic variants are associated with number of neurovascular and psychiatric diseases, which might predispose to stroke and increase its likelihood [23, 24]. Particularly, it was shown that *NGF* rs6330*T minor allele is positively associated with the risk of migraine, schizophrenia and Alzheimer’s disease [13, 14, 25]. As for *NGFR* rs2072446, it was observed as being associated with depression and schizophrenia [13, 15]. Furthermore, inherited rs734194 polymorphism of *NGFR* gene was associated with the decreased risk of obsessive-compulsive disease, schizophrenia and Alzheimer’s disease [13, 22, 26].

According to the data of Cerebrovascular Disease Knowledge Portal most recent GWAS have discovered associations between the studied sequence variants in *NGF* and *NGFR* gene and cerebrovascular disease or related traits. Thus, *NGFR* gene rs2072446 polymorphism was associated with IS of TOAST undetermined etiology (*p* = 0.037), large artery atherosclerosis (*p* = 0.03), height (*p* = 0.01) and waist-hip ratio (*p* = 0.013) [29, 37–40]. Regarding *NGFR* gene rs734194 SNP, its minor allele showed association with TOAST small artery occlusion (*p* = 0.02), Type 2 diabetes (*p* = 0.019), height (*p* = 0.019), serum cystatin C (*p* = 0.016) [39, 41–44]. However, this is the first study to evaluate the association of the genetic variations of the *NGF* and the *NGFR* genes with IS, infarction size and recurrence of the disease in Armenian population. Thus, our findings indicate that mentioned above SNPs could play a potential role in the etiopathogenesis of IS. We suggest that further studies are required to clarify the functional consequences of the mentioned SNPs as well as *NGF* and *NGFR* genes in the etiology and the molecular pathomechanisms of IS. Our results are preliminary and they need to be confirmed in studies with larger sample size and in different ethnic groups.

## Conclusions

Our findings suggest that the *NGF* rs6330*T and *NGFR* rs2072446*T minor alleles might be nominated as a risk factor for developing IS and *NGFR* rs734194*G minor allele as a protective against this disease in Armenian population.

## Abbreviations

CI: Confidence interval
CT: Computer tomography
HWE: Hardy-Weinberg equilibrium
IS: Ischemic stroke
MAF: Minor allele frequency
NGF: Nerve growth factor
NGFR: Nerve growth factor receptor
OR: Odds ratio
PCR-SSP: Polymerase chain reaction with sequence specific primers
SNP: Single nucleotide polymorphism
